# The Life and Works of Harvey
*Harvey on the Circulation of the Blood. Revised and Edited by Alex, Bowie, M.D., C.M. (London: George Bell and Sons.)


**Published:** 1890-08-02

**Authors:** 


### THE PRACTITIONER'S BOOKSHELF.

[Books for Review should be sent to The Editor, The Lodge, Porchester Square, W.]

### THE LIFE AND WORKS OF HARVEY.*

We are never sorry to have our attention once more called to one of the greatest of England's great men, nor to the strangely fascinating study of the way in which the truth after which generation after generation of physiologists were painfully groping, gradually shook off one by one the fetters of pre-conceived ideas, until at last its full glory flashed upon the eager eyes of Harvey. To say that faint glimmerings of the truth had appeared to others, perhaps even as far back as the time of Aristotle, and that others had paved the way for him by discoveries of which they did not apprehend the full value, by no means detracts from Harvey's proper merit. Other observers had collected materials, and even hazarded guesses; his was the master-mind which shook itself free of all trammels, and with fine imaginative insight discovered the principle which was to make all things clear, in a subject which even he, as he tells us, had been once tempted to say, with Fracastorius, "could be comprehended by God alone."

We have only to glance at the theories which were in vogue among the ancients, and which, indeed, had by no means fallen into disrepute with the scientific men of Harvey's time, to see how beset with difficulties was the path of an investigator of the motion of the blood. To say nothing of the fact that the heart was long believed to be the centre of the emotions and passions, physiologists were held in bondage by various metaphysical assumptions, to which facts must be made to fit in—or if not, so much the worse for the facts. Certain spirits or principles were supposed to dominate the body; and as they must needs have free passage somewhere and somehow, it was supposed that the arteries transmitted not only blood, but spirit. There must be, therefore, two kinds of blood—one, the arterial blood, largely mixed with "vital spirits," which were generated in the heart; the other, a dark, venous, "natural" blood, the product, not of the heart, but of the liver. Aristotle had, indeed, spoken of the heart as the source, as well as the reservoir, of the blood; but the "divine Galen" pronounced that the blood was formed in the laboratory of the liver; and so it came to pass that for some 1,500 years physiologists wandered about in a wilderness of error, misled by their fundamental premiss.

To question any dictum of Galen's seemed to the scientific men of the middle ages rank heresy; and those who ran counter to established opinions, whether in science or religion, must risk, not only reputation, but property, freedom, and even life itself. Servetus, a man of bold speculative spirit and inductive genius, was forced to live under an assumed name, and at last perished miserably at the stake, the freedom of his opinions having incurred the wrath of the stern Calvin. In his work on "The Restoration of Christianity," he did actually suggest (a curious instance, by the way, of the general hotch-pot into which the thinkers of those days cast their ideas) that the blood from the right side of the heart traversed the lungs before entering the left side, though it does not appear that he had before him any anatomical evidence of the truth of his theory. Cæsalpinus, again, whose "Peripatetic Questions," like Servetus' work, treated of religious and ecclesiastical, as well as physiological, matters, only escaped censure and probable ruin by entirely disclaiming for his own part the conclusions to which he had declared the Aristotelian philosophy to lead. He certainly seems to have observed that the blood moves in but one direction in the veins, and even makes use of the word "circulation" with reference to its movement; but there can be no doubt that he did not understand by this term what Harvey understood by it. Still, we may freely admit it to be possible that either he, if he had been less trammelled by the authority of Galen—or Servetus, if he had not been cruelly cut off in the prime of life—might have anticipated the great discovery made some fifty years later.

Harvey himself was evidently apprehensive of the treatment which he might receive as the promulgator of a theory running counter to those of long-recognised authorities; for although he seems to have lectured upon his new doctrine as early as 1616, it was not till 1628 that his "Exercise on the Motion of the Heart and Blood in Animals" first saw the light. And, indeed, his friend Aubrey tells us that after its appearance "he fell mightily in his practice. 'Twas believed by the vulgar that he was crack-brained, and all the physitians were against him." Certainly there were not wanting men who bitterly, and, indeed, viciously, attacked both himself and his doctrine; but he steadily declined to return railing for railing, quietly observing that "it cannot be helped that cynics should be numbered among philosophers"; and no doubt his dignified self-restraint made the conflict less bitter than it would otherwise have been. On the whole, we think that, in comparison with other discoverers of truth, he may be said to have fared very well. Although his practice may to some extent have fallen off, the sum of money he left behind him proves that his emoluments must have been large; he remained Court physician for many years after the publication of his great work, and he enjoyed the friendship of the most eminent men of the day, among them the illustrious Bacon, whom, oddly enough (considering that he himself pursued the inductive method), he "esteemed much for his witt and style, but would not allow to be a great philosopher." But what Harvey doubtless valued most was the esteem of his medical brethren, unmistakably shown by their pressing upon him, some three years before his death, the Presidency of the College of Physicians; and the gradual recognition by the scientific world, continental as well as English, of the doctrine which at first met with such bitter opposition. His antagonists, Riolan and Caspar Hoffman, indeed, remained unconvinced to the end of their days, though Schlegel (why does Dr. Bowie call him Slegel, by the way?) had hopes that the latter would have given way, had he only lived a little longer. But Dionis, Riverius, Descartes, Bartholin, Walæus, and many others, became ardent supporters of the new doctrine; and before Harvey died, he had the joy of knowing that his discovery was generally received. Could he but have lived to see, with Malpighi, by the aid of the magnifying glass, the communication between arteries and veins whose existence he had been convinced of, but had been unable to prove by ocular demonstration, there had been nothing wanting to his triumph.

* Harvey on the Circulation of the Blood. Revised and Edited by Alex. Bowie, M.D., C.M. (London: George Bell and Sons.)

